# Management of progressive type 2 diabetes: role of insulin therapy

**DOI:** 10.1186/1750-4732-3-5

**Published:** 2009-07-02

**Authors:** Ramachandra Rahul V Chemitiganti, Craig W Spellman

**Affiliations:** 1 Texas Tech University Health Science Center, 701 West 5th Street, Odessa, TX 79763, USA

## Abstract

Insulin is an effective treatment for achieving tight glycemic control and improving clinical outcomes in patients with diabetes. While insulin therapy is required from the onset of diagnosis in type 1 disease, its role in type 2 diabetes requires consideration as to when to initiate and advance therapy. In this article, we review a case study that unfolds over 5 years and discuss the therapeutic decision points, initiation and advancement of insulin regimens, and analyze new data regarding the advantages and disadvantages of tight management of glucose levels.

## Introduction

Diabetes currently affects 23.6 million people in the United States [1,2] and 246 million people worldwide, and it is expected to increase to 380 million by 2025 [3]. In the United States, type 2 diabetes (T2D) accounts for 90% of all newly diagnosed cases. There are 57 million Americans who have prediabetes and 5%–7% of these people progress to T2D each year [1]. People with T2D have an increased risk for vascular complications, most of which can be prevented with aggressive management of the metabolic disturbances that are associated with the disease.

Data from the National Health and Nutrition Examination Survey (NHANES) demonstrate that the proportion of adults in the U.S. with diagnosed and adequately controlled T2D increased significantly between 1999–2000 and 2003–2004. Overall, only 37% of the adults in NHANES 1999–2000 had HbA1C (A1C) levels at the American Diabetes Association (ADA) goal of < 7% and only 36% had a blood pressure of 130/80 mm/Hg, and just 50% had a total cholesterol < 200 mg/dL, while in the 2003–2004 study 56.8% of patients reached a goal A1C of <7% [4,5]. Thus, only half of the people with T2D are adequately managed for glucose control and overall only 7% are at goal for blood pressure, lipids and glucose [4].

Implementing clinical guidelines from the American Diabetes Association [6], the American Association of Clinical Endocrinologists [7] and understanding the newer consensus statement from the American Diabetes Association and the European Association for the Study of Diabetes [8] and recommendations from the Texas Diabetes Council [9] could help explain the decrease in A1C levels in recent years.

The following case presentation will exemplify chronologically the onset of T2D and therapeutic decisions that are involved in prevention and delay strategies, use of oral agents, incretin-based therapies, and transition to insulin management.

## Case presentation

*Ruth was a young woman, age 26 years, when she was first seen as a new patient in the clinic. She worked at a nightclub and had earned some college credits toward a degree in elementary education. Although she had no known health problems, she had questions and concerns about her risks for diabetes and wanted some screening tests performed. Both of her parents had T2D and developed renal complications. At the visit, Ruth weighed 134 lbs and was 5'4" tall (BMI = 23 kg/m*^2^). *Baseline screening studies revealed her fasting glucose to be 86 mg/dL and liver function to be normal. Lipids showed triglycerides 213 mg/dL, high-density lipoprotein cholesterol (HDL-C) 38 mg/dL, and low-density lipoprotein cholesterol (LDL-C) 136 mg/dL*.

**Question 1**: What would you tell Ruth about her risk of developing T2D and can it be prevented?

**Answer**: Impaired glucose metabolism is common in first-degree relatives of patients with T2D. Offspring of these patients have a 30%–40% chance of developing T2D [10] and frequently demonstrate abnormal glucose tolerance and several associated metabolic abnormalities such as low insulin release or insulin resistance [11-13].

Thus, although her glucose is currently in the normal range, keeping in mind her family history, Ruth probably would have a tendency to develop insulin resistance. The best strategy at this point is to recommend lifestyle changes that include nutrition therapy and regular physical activity. Because of the effects of obesity on insulin resistance, weight loss is an important therapeutic objective in overweight or obese individuals [14]. Simple attention to lifestyle has been demonstrated to be superior to medications for the prevention and delay of T2D in several studies [15-19]. Furthermore, this approach will have a positive impact on the lipid abnormalities [20].

Short-term studies have shown that moderate weight loss (~5% of body weight) in T2D patients is associated with decreased insulin resistance, improved glycemic control, and improved measures of lipemia and blood pressure [15]. Intensive lifestyle intervention employed in the diabetes prevention program (DPP) and maintenance of weight loss at ~5% over 3 years led to a 58% reduction in the incidence of T2D [16]. One-year results from the look AHEAD (Action for Health in Diabetes) study demonstrated that intense lifestyle intervention resulted in an average of 8.6% weight loss, reduction of A1C, and reduction of several cardiovascular disease risk factors [21].

Since Ruth's BMI (23 kg/m^2^) is currently normal, based on the ADA guidelines [6], it was recommended that she perform at least 150 minutes/week of moderate-intensity aerobic physical activity. Resistance training three times per week is an alternative.

*Subsequently, Ruth disappeared from the clinic for 3 years. When she returned, life had taken some different turns. Two years ago she had become pregnant and developed gestational diabetes. Diet and exercise were successful for managing the glucose abnormality and her baby did well. However, physical examination now showed that her weight was 174 lbs (BMI = 29.9 kg/m*^2^) *and a random blood sugar was 208 mg/dL*.

**Question 2**. Do you think Ruth now has T2D?

**Answer**: Ruth may have T2D, but the formal diagnosis is based on observing an abnormal glucose and then confirming the glucose abnormality. That is, two tests are needed to make the diagnosis. The gold standard for diagnosing T2D is still the oral glucose tolerance test. It is more sensitive than the simplified methods of measuring fasting glucose on two occasions or finding a random glucose ≥200 mg/dL in a symptomatic person [6].

There is good reason to recommend that Ruth have a glucose challenge: First, we already know that she is genetically predisposed to develop T2D, and she has gained 40 pounds. Second is the fact that she developed gestational diabetes: Ruth has failed the endocrinologist's "stress test" in that she was unable to handle the increased metabolic demands of pregnancy 2 years ago, and gestational diabetes is a very high risk factor for subsequent T2D. Overall, almost 50% of Caucasian and up to 80% of Hispanic women who have had gestational diabetes will progress to T2D within 5 years [22-25].

*Ruth was shocked when told she might have diabetes and that confirmatory tests were needed. However, she declined further testing at this time and said she was going to get serious about losing weight and then do the tests. Three months later when she returned her weight was still 172 lbs*.

**Question 3**: If a subsequent oral glucose tolerance test is positive for T2D, does Ruth need further evaluations to differentiate type 1 diabetes from T2D?

**Answer**: Based on Ruth's history of gestational diabetes, weight gain, her family history of diabetes, and the rate of onset of hyperglycemia it is very likely that she has T2D. Specialized tests are not indicated for this patient. Testing for insulin C-peptide, anti-islet cell (ICA), and anti-insulin (IAA), or antiglutamic acid decarboxylase (GAD) antibodies is a matter of clinical judgment. These studies may be considered in *non-obese *adults who present with a T2D-like picture and for whom it is unclear whether the patient is catabolic [26]. An estimated 20% of such patients thought to have T2D actually have latent autoimmune diabetes or type 1 diabetes. These patients will require (or soon will require) an insulin-based therapeutic regimen. Although the antibody panel helps to differentiate T2D from latent autoimmune diabetes, it must be emphasized that these tests are not diagnostic for the latter. However, the picture is different in pediatric cases of diabetes. Pediatric endocrinologists often obtain autoantibody markers and C-peptide levels in children presenting with hyperglycemia [27].

*Ruth proceeded to have an oral glucose tolerance test and the diagnosis of T2D was confirmed. Further baseline studies revealed normal renal and hepatic function, the urine microalbumin to creatinine ratio was 6 mcg/mg (normal <20), and the A1C was 6.6%. She had already started maintaining self-monitored blood glucose (SMBG) records. A review showed the fasting glucose to average 120 mg/dL and the 2-hour postprandial values averaged 180 mg/dL. Ruth knew what the values in the SMBG records meant and was overwhelmed at the prospect of lifelong treatment of diabetes*.

**Question 4**: What therapy options would you recommend to Ruth?

**Answer**: Diet, exercise, and weight control always remain the cornerstones of any therapeutic plan in T2D. In addition, Ruth needs to begin monotherapy with an oral antidiabetic drug. Monotherapy is appropriate in a clinical setting where A1C levels are ≤7% with no evidence of micro- or macrovascular complications. At this stage, any oral antidiabetic medication (thiazolidinediones, dipeptidyl peptidase-4 inhibitors, alpha glucosidase inhibitors, biguinides, glinides or sulfonylureas) can be considered appropriate. However, metformin is generally recommended as the initial pharmacological agent in those with normal renal function [8]. Also, insulin is always an option for initial therapy [8,9].

Some physicians believe that patients gain much weight if prescribed a thiazolidinedione (TZD) rather than an alternative therapy. However, data reveal this is not true. Depending on the source, weight gains of about 1 to 3.6 kg are seen with TZD-managed patients who follow a diet and exercise plan [28,29]. Large weight gain occurs when patients begin using a TZD and do not change their eating habits. Some patients (5%) taking a TZD will develop edema and it is well appreciated that TZDs should be avoided in patients with decreased ventricular function (New York Heart Association class 3 or 4 failure) [30]. Although the FDA still recommends analysis of liver enzymes every 3 months for the first year of TZD therapy [31], it should be noted that the newer TZDs, rosiglitazone and pioglitazone, have not been associated with transaminitis.

Numerous studies have shown that some sulfonylureas are associated with higher risk of hypoglycemia [32-34]. However, glipizide and glimepiride are considered safer because they are associated with less hypoglycemia [35].

*Ruth began using metformin and the dose was titrated upward. However, after 3 months, glycemic control remained suboptimal. The fasting glucose averaged 145 mg/dL and her mean 2 hr postprandial levels were 220 mg/dL. The A1C study was updated and found to be 7.1%*.

**Question 5**: What would be your next recommendation to Ruth for achieving glycemic control?

**Answer**: Ruth needs combination therapy. Two-drug regimens are indicated in persons with an A1C of 6.5% to <8% if they are already on monotherapy. Incidentally, combination therapy is indicated as initial therapy for treatment-naïve patients with an A1C of 7% to 8% [7]. Combination therapy may include any two oral agents, as long as each drug is from a different class. It would make no sense to place a patient on two sulfonylureas or two TZDs. One might also consider adding an incretin mimetic such as exenatide or liraglutide to the monotherapy program if the A1C was ≤1% above goal. Again, insulin is an option at any A1C level [7].

*Ruth opted to combine sitagliptin, a DPP-4 inhibitor, with the metformin as the next step to control her diabetes. Her glycemic control improved markedly and she was very pleased that the A1C remained around 6.5%. However, laboratory studies a year later demonstrated progression of her diabetes. The A1C had increased to 7.8%. Her BUN, creatinine, and hepatic functions remained normal, but the urine microalbumin to creatinine ratio was now abnormal at 30 mcg/mg. Her SMBG records were roughly consistent with the A1C; the fasting average was approximately 155 mg/dL and the daytime glucose averaged 170 mg/dL. Combination therapy with two oral agents was no longer effective*.

**Question 6**: What is the next step? Should you add a third pill? Add an incretin mimetic? Start insulin?

**Answer**: Clinical judgment must come into play and be weighed with the patient's wishes on escalating therapy. The A1C is 7.8% and this places us on the cusp for several appropriate clinical decisions. Some physicians might add a third oral agent, some might recommend exenatide, and still others would initiate insulin. The rationale for selecting the next therapy can be simplified by using the following maxim: In general, each oral agent alone can reduce the A1C by 1.0%–2.0%, but each added oral agent results in further A1C reductions of about 1% [36]. Exenatide will usually reduce the A1C about 1% [37]. Thus, adding an additional pill or incretin mimetic to an existing regimen may be effective if the A1C is ≤1% above goal. If the A1C is >1% above goal, then adding a third agent may not be an effective strategy. Such patients would probably need insulin. If insulin is selected as the next step, many physicians stop or decrease the sulfonylurea. At this time, exenatide and sitagliptin are not approved for use with insulin. If the patient is taking metformin and/or a TZD, most physicians would continue these because they are, in effect, "insulin sensitizers."

In the current case study, when Ruth's glucose control slipped, and A1C levels increased to 7.8%, we notice the incidence of microalbuminuria, which is a major risk factor for renal and cardiovascular events. The Heart Outcomes Prevention and Evaluation Study demonstrated that microalbuminuria increased the relative risk for major cardiovascular events by 1.83-fold and hospitalization for heart failure by 3.23-fold [38]. There is a wealth of data that show significant risk reductions for incident microalbuminuria in individuals treated to achieve tight glycemic control. Data from the BENEDICT trial [39] demonstrated that the risk of developing microalbuminuria can be effectively reduced.

*Although reluctant to begin insulin, Ruth came to the conclusion that an insulin regimen was probably the best approach. She considered the rate at which her diabetes had progressed and the value of achieving tight control. She remembered her parents and their downhill course with kidney failure. She also remembered what she learned about tight glucose control and the remarkable reductions in microvascular disease. So, although a third oral agent and exenatide were viable options, she decided to start insulin. She was most excited to discover that insulin injections were almost "trivial" with the pen systems and micro needles and eliminated the inconvenience of carrying insulin vials and syringes. There is also data demonstrating that using pen devices may result in better glycemic control *[40,41].

At the first return visit to review glycemic control and titrate the insulin dose, Ruth reported that "If I shoot a stretch mark, I absolutely can't feel anything!" She was reminded that insulin absorption was altered in scar tissue and that she should use a different site on the abdomen. She declined to change and said that she had now grown to love her "ugly stripes."

**Question 7**: How is insulin therapy initiated?

**Answer**: The goal of insulin administration in patients with diabetes is to mimic normal physiologic secretion of insulin to control both fasting plasma glucose (FPG) and postprandial glucose (PPG) levels. There are several ways to start insulin as long as the dose and timing are appropriate. The challenge is to actually start insulin when it's indicated and not delay therapy until end-organ problems surface. Insulin should be started within 3–6 months if combination therapy cannot achieve A1C goals [20].

Historically, regular human insulin (RHI) was used to control PPG and the intermediate-acting neutral protamine Hagedorn (NPH) was used to control basal glucose. These remain effective insulin preparations, but the newer insulin analogs are replacing RHI and NPH. Both RHI and NPH exhibit considerable intra- and interpatient variability in absorption [42-44]. Aspart, glulisine or lispro are preferred by many patients over RHI because lifestyle flexibility is afforded due to their rapid absorptions and short duration of action [44]. For example, in contrast to RHI, which must be administered at least 30 minutes before a meal and has an effective duration of 4 hours, rapid-acting insulin analogs can be administered within 15 minutes before eating, at the start of a meal or even after a meal and its effects wane within 2 hours [45]. The main reason detemir and glargine are used over NPH is because the risk of hypoglycemia, in particular nocturnal hypoglycemia, is decreased [46,47].

A patient may start by using 10 units of insulin or 0.10 to 0.25 units insulin per kg on a once-daily regimen [48]. These starting doses are appropriate for glargine, detemir, NPH, premixed 70/30 aspart-protamine/aspart or 75/25 lispro-protamine/lispro. Glargine or detemir should be started with a once-daily injection. NPH is often started with a bedtime injection. Premixed insulin may be started pre-breakfast and/or pre-dinner [49,50]. As noted above, the oral agents are continued except for adjustments if a sulfonylurea had been used. Exenatide should be discontinued because study results are not yet available on its use with insulin.

The old rule for starting insulin was simple and the goal was to control the fasting glucose first because that set the tone for the entire day. The insulin dose was increased every 2–3 days in 2- to 3-unit increments until the fasting glucose is controlled [48,51]. The second goal was then to control the daytime glucose. The old rule works fine if the patient is still deriving benefit from the oral agents. Specifically, once-daily insulin is sufficient if control is achieved *and *the fasting glucose is approximately the same as the bedtime glucose. Why? We will return to this point and explain it in our discussion of question 9, below.

*Ruth started using 10 units basal insulin every morning and titrated the dose herself for 2 weeks. At the return visit, she was using 18 units and was unhappy because "my sugars are still crappy." Her SMBG records showed the fasting glucose to average 124 mg/dL, but they were elevated to 160 mg/dL before meals and 230 mg/dL 2 hours postprandially. Her first questions concerned whether she was on the right insulin*.

**Question 8**: What are Ruth's options for glucose management?

**Answer**: Ruth requires either multidose or intensive insulin therapy [36]. The fasting glucose had improved, but the daytime glycemic excursions were not controlled with oral agents and basal insulin alone. One option is to advance directly to intensive therapy with basal glargine or detemir each morning and prandial aspart, glulisine, or lispro with meals. A second option is switching to premixed insulin. Again, selection of insulin regimens needs to be done with considerations of the patient's life schedules. Basal-prandial schedules involve more injections, but there is more freedom. That is, the prandial insulin is used only when and if the patient eats. Premixed insulin, which contains intermediate and fast-acting insulin, can be used twice or three times daily and requires fewer injections. However, the patient must adhere to a daily dietary and activity schedule.

*Ruth opted to switch to a twice-daily premixed insulin regimen. She wanted to give this approach "a few months to see how well it works." She again stressed that her goal was to control the sugars. After 6 months, her A1C was 6.4% but she had difficulties adjusting her schedule on weekends and eating on time. She was happy with her results, but was also eager to try basal-prandial therapy to determine if it would better fit her lifestyle*.

**Question 9**: How are insulin regimens changed?

**Answer**: Ruth had achieved good control using premixed insulin. She had started twice-daily premix based on her previous dose of basal insulin. She simply divided the old dose into two injections, 10 units morning and 10 units in the evening, and titrated up to 25 units each AM and 15 units each PM to achieve control [52]. Now, the question arises as to the best approach for converting from a twice-daily premixed regimen to a basal-prandial regimen. Only one method will be presented here [53]. It should be realized that the following strategy will work for any question on conversion from a multidose schedule to a basal-prandial regimen. It doesn't matter if the conversion is from premixed insulin or from NPH plus regular, aspart, glulisine or lispro insulin.

Do this: Simply stop the current therapy and start over with basal and prandial insulin. The basal insulin should be glargine or detemir. The prandial insulin should be aspart, glulisine, or lispro. Because the patient has been on insulin the weight-based dose is now 0.5 units per kg [52]. (Remember, the patient who was insulin-naïve starts at 0.10 to 0.25 units per kg insulin.) Half of the insulin dose is given once daily as basal insulin and the other half is given as aspart, glulisine, or lispro divided among the three meals. If Ruth weighs 170 pounds (or 77 kg), about 38 units will be needed per day. Thus, she will take 19 units of daily basal insulin and about 6 units of prandial insulin before each meal. Basal insulin is titrated, as before, by increasing the dose 2–3 units every 2–3 days until control of the fasting glucose is attained. The prandial insulin is titrated in a similar fashion: Each dose is individually adjusted by increasing 1–2 units every 2–3 days until each postprandial value is <140 mg/dL (AACE) or <180 mg/dL (ADA) [54]. An important point to stress at this time concerns the ratio of basal to bolus insulin. The guiding principle is that the basal-prandial regimen should approximate physiologic insulin delivery. This means that approximately 50% of the body's insulin requirements are for management of basal metabolism. Approximately 50% of the body's insulin requirements are for disposal of nutrients. The 50:50 ratio is not an iron-clad rule, but serves as a good starting point for insulin titrations. Some persons may require 60% basal and 40% prandial. During pregnancy, 40% basal and 60% prandial use is not uncommon. At this point, we are going to explain my statement in Question 7 about why the fasting and bedtime glucose values should be approximately equal and give you an important tip on titrating insulin: Aim for the fasting blood glucose to be about the same as the blood glucose measured at bedtime! Basal insulin is used to control hepatic glucose production. It's not intended to shut-off hepatic glucose production. Basal insulin is not intended to compensate for an insufficient dose of prandial insulin. If the patient's bedtime blood sugar was 220 mg/dL and the fasting glucose is 95 mg/dL, you are using too much basal insulin and not enough supper time prandial insulin. It's also a good bet that the patient with high bedtime sugars and "well-controlled" AM sugars probably had an unrecognized hypoglycemic event at sometime during the night.

**Question 10**: What if Ruth had opted to stay on basal insulin, not start premix, and needed to add prandial coverage?

**Answer**: Ruth was approaching the fasting glucose goal but the daytime sugars were high. To address this, aspart, glulisine or lispro would have been added to each uncontrolled meal time. The starting dose of prandial insulin is 5 units or 0.1 units/kg and each dose of prandial insulin is titrated as above to achieve the 2-hour postprandial goals. Here is the tip we teach residents: When adding prandial insulin to an ongoing basal regimen, decrease the basal dose about 10% for each added prandial dose. Then, don't forget that during this titration period, you want the bedtime and fasting glucose to be the same. Once achieved, you can then begin fine tuning the regimen.

*Over the next year, Ruth did well. Her A1C remained between 6.5 and 7%, the urine microalbumin to creatinine ratio decreased to 8 mcg/mg, and other markers for renal and hepatic function remained normal. On a recent follow-up visit to review glycemic control she had questions about the safety of insulin therapy. "I heard on the news and everywhere else that insulin caused low sugars and death. Should I really be taking it?" Ruth was referring to the headlines on the ACCORD Trial *[55]. *This is a common scenario*.

**Question 11**: How can we answer Ruth's question about whether she should continue on insulin therapy?

**Answer**: Let's start by asking why the NIH-sponsored ACCORD Trial was done? The rationale was that the major world studies, including the UK Prospective Diabetes Study [56] did not conclusively demonstrate that tight glucose control in T2D would reduce the incidence of cardiovascular disease. Thus, ACCORD was designed to determine if glucose management in the setting of controlled blood pressure and lipids would reduce macrovascular events. Approximately 10,000 people were recruited, and it is important to know their characteristics. The participants had an average age of 62 years, had long-standing type 2 diabetes, about 1/3 had a known previous cardiovascular event and 2/3 had multiple risk factors. Standard treatment targeted an A1C of 7%–7.9% and maintained an average of 7.5%. Intensive treatment targeted an A1C of <6% and maintained an average of 6.4%. The intervention was insulin therapy added to any diabetes control regimen with oral agents or exenatide. So what was found after 3.5 years? The intensive therapy arm of the trial was halted 18 months early. The review cited increased death in the intensive treatment group, possibly related to hypoglycemia. What made the headlines? Intensive insulin therapy for diabetes causes death.

However, the ACCORD data can be looked at in several ways. The data did not demonstrate that glycemic control reduced macrovascular disease but the question is still considered unanswered by many. Was the increment change in A1C between intensive and standard therapy large enough to detect changes in macrovascular outcomes? Would the impact of glycemic control surface if the study continued for a longer time? What if emphasis on glycemic control had been implemented earlier in the disease course?

From another vantage point, the ACCORD data show very positive results after controlling blood pressure, lipids and glucose. Let us use the information from Hafner's study of mortality from coronary heart disease in diabetic and nondiabetic subjects with and without a prior history of myocardial infarction, and whose blood pressure, lipids and glucose were not controlled [57]. Sixty-two percent of the participants had hypertension; the average blood glucose was approximately 200 mg/dL and the average LDL cholesterol was170 mg/dL. Looking at Figure one in Hafner's report, at approximately 3.5 years into the study the death rate was about 3% per year in those with diabetes (with or without a prior history of myocardial infarction.) Now we see the value of treating hyperglycemia, hyperlipidemia and hypertension. The observed death rates in the ACCORD were 1.1% with standard therapy and 1.4% in the intensive therapy group. If we express the data in terms of death per thousand patients per year, cardiovascular death in untreated diabetes is 30/1000 *versus *11/1000 and 14/1000 in the ACCORD standard and intensive insulin therapy groups, respectively. Both standard and intensive insulin therapy groups showed benefit! It's true that the death rate between intensive and standard therapy was statistically significant (3/1000 patient-years) and hence the intensive intervention was appropriately stopped, but here are the clinical implications. First, controlling glucose, blood pressure and lipids is effective in preventing cardiovascular death in patients with diabetes, although the contribution of glycemic control is unresolved. Secondly, an A1C of 7.5% appears to be acceptable for older patients with long-standing diabetes and multiple cardiovascular risk factors or disease.

Further support for glucose management in the setting of controlled blood pressure and lipids comes from the ADVANCE study [58] which was published in the same journal with the ACCORD. Similar reductions of major CV events and overall mortality were reported with intensive and standard insulin therapy. However, unlike the ACCORD results, no excess death was found with intensive insulin management.

To answer Ruth's question, one can review the following points. She is 31 years old and not in the 60+ year old category. Her A1C is about 6.5% without adverse events or hypoglycemic episodes. Her urine microalbumin to creatinine ratio has improved and insulin was needed to achieve this degree of control when oral agents had failed.

There are 3 significant take-home messages:

1. Insulin therapy, whether a once-daily regimen with basal insulin or premixed insulin, is appropriate as an initial therapy for type 2 diabetes.

2. Insulin therapy should be started when the A1C is approximately 1% above goal in patients on combination therapy and must be advanced to a multidose or basal-prandial regimen if fasting and/or daytime glucose is not at goal.

3. Insulin therapy is beneficial, not deleterious, in the management of diabetes.

## Competing interests

The authors declare that they have no competing interests.

## Authors' contributions

CS conceived the manuscript. The manuscript was written by CS and RC. Both CS and RC have read and approved the final manuscript.
